# A Comprehensive Review of Pharmaceutical and Surgical Interventions of Prostate Cancer

**DOI:** 10.7759/cureus.11617

**Published:** 2020-11-22

**Authors:** Jasndeep Kaler, Azhar Hussain, Ayema Haque, Hassan Naveed, Sundip Patel

**Affiliations:** 1 Medicine, Xavier University School of Medicine, Oranjestad, ABW; 2 Healthcare Administration, Franklin University, Columbus, USA; 3 Internal Medicine, Dow University of Health Sciences, Civil Hospital Karachi, Karachi, PAK; 4 Internal Medicine, St. Matthew’s University School of Medicine, Grand Cayman, CYM; 5 Medicine, Windsor University School of Medicine, Cayon, KNA

**Keywords:** prostate-specific antigen, prostate cancer, androgen deprivation therapy, prostatectomy, turp, gleason score

## Abstract

As the second most common cause of death amongst men in the United States, prostate cancer is a type of cancer that is known to develop and originate in the prostate gland. The main function of the prostate gland is to produce seminal fluid in which the sperm bathes. The seminal fluids are necessary for allowing the sperm to move easily through the urethra and also allows successful fertilization by providing an alkaline environment for the sperm in the acidic nature of the vagina. The seminal vesicles are two smaller glands that are attached to either side of the prostate gland and in radical prostatectomies, can get removed. In the event that the seminal vesicles are removed during a radical prostatectomy, the individual is unable to produce any seminal fluids and thus, becoming infertile. Prostate cancer is most commonly seen in patients over the age of 66 years, however, in the presence of predisposing risk factors, may occur as early as in the late 40s. Certain risk factors may speed the presentation of prostate cancer in individuals and thus, mandatory screening is recommended around the age of 45. If no risk factors are present, screening is recommended to begin after the age of 50 years. Screening for prostate cancer is focused on looking for prostate-specific antigen (PSA) in a blood test, though this may not be the most reliable method. The method of diagnosis stems from further testing done following an abnormal PSA test. A digital rectal examination and ultrasonography may also be used to assist with the diagnosis of prostate cancer. Though there are several different types of pharmaceutical interventions currently present in the eradication of prostate cancer, with androgen deprivation therapy being the most commonly used, surgical interventions may be utilized to completely resect cancer from an individual. Different radical prostatectomies are used; the appropriate approach utilized is dependent on the extensiveness of cancer and the type of cancer that is present.

## Introduction and background

Prostate cancer is one of the most common, noncutaneous cancers among men in the United States [1,]. Being the second-leading cause of death among men in the United States, about one in nine men will be diagnosed with prostate cancer in their lifetime [3]. Despite the high incidence rate of prostate cancer, relatively few patients with prostate cancer die of the disease. This still averages to be a little over 26,000 deaths per year in the United States [4]. The incidence of prostate cancer surely varies globally, however, the highest rates of incidence are found in the United States, Canada, and Scandinavia [5]. Located in the male pelvis at the base of the penis, the prostate gland’s function is to produce about a third of the total seminal fluid [6]. The seminal fluids function to nourish and transport sperm through the seminal vesicles, which are two smaller paired glands that are attached to either side of the prostate gland. According to data from the National Cancer Institute, there is an 11% risk of being diagnosed with prostate cancer for an average American male [7], and this risk is known to increase with age. While the risk of prostate cancer increases with age, the aggressive nature of the cancer is known to decrease [6]. With no early symptoms, the diagnosis of prostate cancer is primarily based on transrectal ultrasound-guided prostate biopsies and prostate-specific antigen (PSA) testing [6,7]. Though the screening of prostate cancer is highly recommended for all men after the age of 50 years, for those with predisposing risk factors such as family history, screening is recommended to begin earlier, around the age of 45. With age and family history being the primary contributing risk factors to the development of prostate cancer, other noted risk factors have been ethnicity, diet, and hormonal factors [5]. Age drastically increases the risk of prostate cancer under the age of 40, prostate cancer being rare but being very common over the age of 65 years [8]. The primary reason for any type of screening is to detect any premature stages of prostate cancer and as such, allowing for earlier intervention to help prevent unnecessary morbidity and mortality [9].

As mentioned earlier, the prostate gland plays a pivotal role in the male reproductive system. The primary role of the prostate gland is to secrete an alkaline solution to protect sperm in the acidic environment of the vagina [7]. The alkaline solution allows the sperm to survive the acidic environment of the vagina, thus, increasing the chances of successfully fertilizing an egg. Moreover, secretions from the prostate gland also contain supportive proteins and enzymes that function as providing nourishment to the sperm [7,8]. The prostate, in itself, is a dense fibromuscular gland that lies directly inferior to the bladder and wraps around the proximal urethra in the lesser pelvis [8]. Prostate cancer is most common in the peripheral zone which is the portion of the prostate gland palpable during a digital rectal examination (DRE) [5]. A DRE is able to note any surface abnormalities such as asymmetry, the presence of any hard nodules however, DRE is not considered to be definitive. An abnormal DRE initially only uncovers about 20% of all prostate cancers [6]. This review aims to identify the different subtypes of prostate cancer, the method of diagnosis, and the surgical and pharmaceutical treatment options for prostate cancer.

## Review

Discussion 

**Figure 1:**
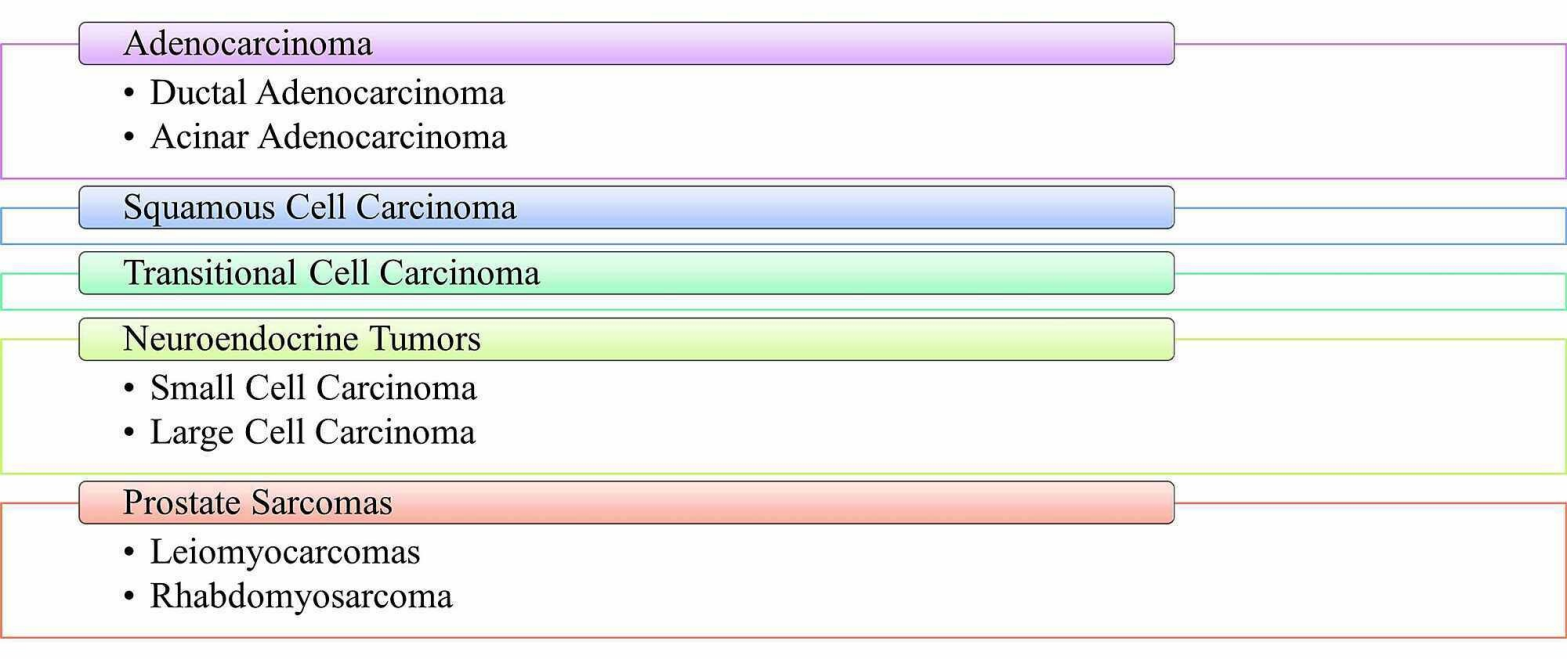
The different types of prostate cancer

Figure 1 is a depiction of the various types of prostate cancer. Prostate cancer develops when the rates of cell division and cell death are no longer at equilibrium, and thus, the imbalance leads to uncontrolled tumor growth. With only 1-5% of prostate cancers being other types, the most common type of prostate cancer is adenocarcinoma [10]. Adenocarcinoma of the prostate develops from the glandular cells responsible for making up the prostate fluid and can be further divided into acinar adenocarcinoma and ductal adenocarcinoma. Between the two subtypes of adenocarcinoma, acinar adenocarcinoma is more common. In acinar adenocarcinoma, the mutated rapid growth of cancer cells begins in the prostate's peripheral zone, making it one of the types of prostate cancer that the physician may feel during a DRE [11]. On the contrary, ductal adenocarcinoma is less common and typically occurs in conjunction with acinar adenocarcinoma [10,11]. With ductal adenocarcinoma, the cancer cells are more centralized in the prostate and are more aggressive than acinar adenocarcinoma [11]. Transitional cell carcinoma, also known as urothelial cancer, typically starts in the structures surrounding the prostate, including the cells lining the urethra [10]. Transitional cell carcinoma is typically diagnosed only after symptoms appear, with difficulty urinating is the most common. This is largely due to this type of subtype beginning in the urethra and spreading to the prostate.

Neuroendocrine tumors are also referred to as carcinoid tumors. Typically, these types of tumors are present in the neuroendocrine system and are responsible for making and releasing hormones into the bloodstream, such as serotonin [10,11]. Neuroendocrine tumors are slow in growth and most often start in the gastrointestinal (GI) tract before moving elsewhere, such as the prostate [10]. Due to the sporadic nature of the prostate gland's neuroendocrine tumors, not much information is available concerning its exact origin and what causes the development. What is known is that neuroendocrine tumors of the prostate do not affect the PSA levels. Because neuroendocrine tumors secrete hormones, such as serotonin, an individual may have increased hormonal levels. Although neuroendocrine prostate cancers are rare, the most common is an aggressive type called small cell prostate cancer, as shown in Figure 1 [11]. The small cell carcinoma subtype of neuroendocrine tumors is very aggressive and has typically metastasized when discovered [10,11]. Sarcomas, on the other hand, usually develop within the soft tissues such as muscles and nerves [10]. Because sarcomas develop in the soft tissues, they can develop anywhere and travel anywhere. The two most common prostate sarcomas are leiomyosarcomas and rhabdomyosarcoma, affecting younger men between the ages of 35 and 60 [10-11]. Because prostate sarcomas comprise less than 0.1% of all prostate cancers, they can usually be challenging to detect and because PSA levels do not change in response to these tumors. Squamous cell carcinoma is another fairly rare type of prostate cancer that is also fast-growing and is very aggressive [10].

Risk Factors

While investigating the different subtypes of prostate cancer, what remained constant was the presence or absence of risk factors.

**Figure 2:**
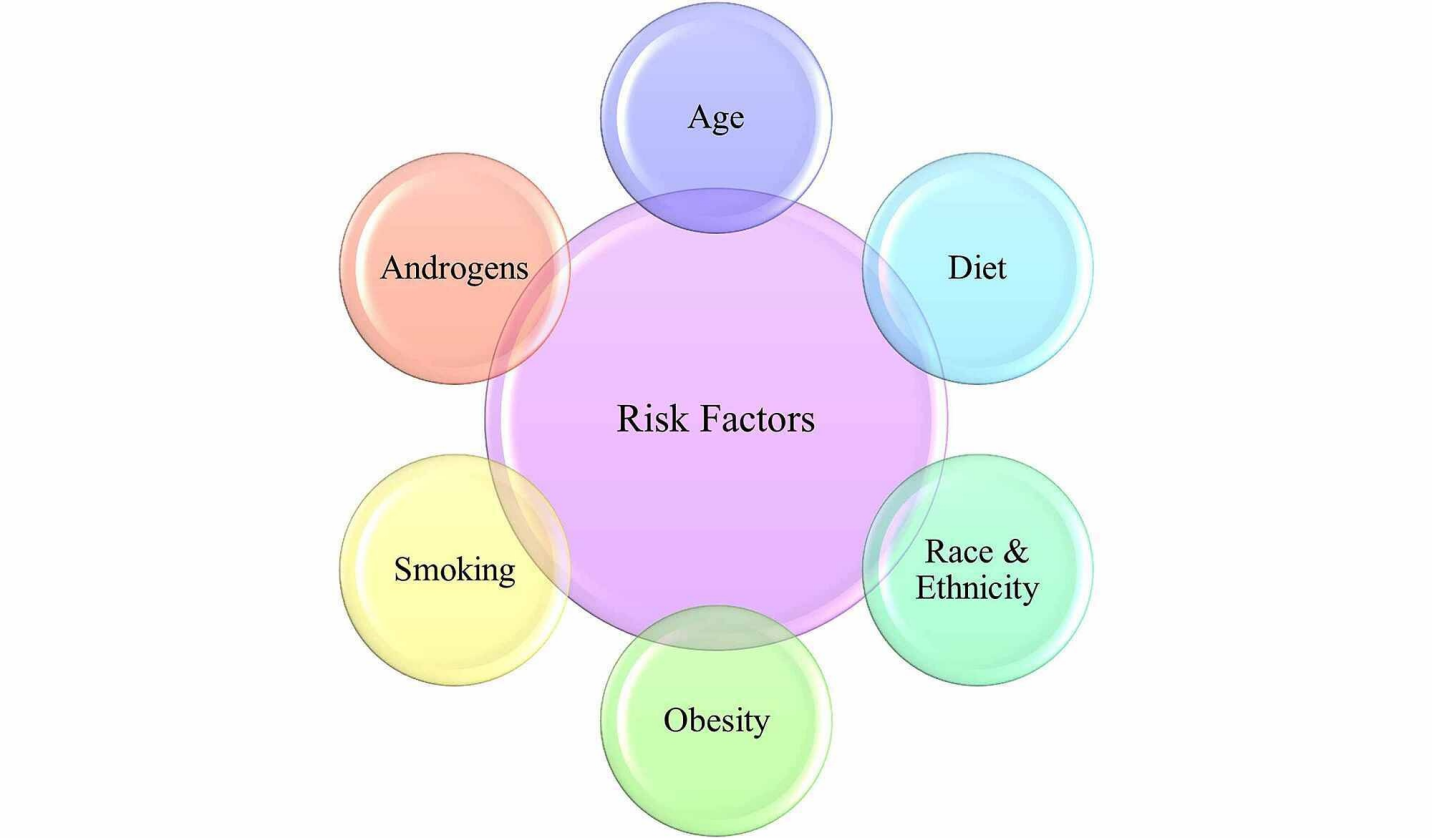
Risk factors of prostate cancer

Figure 2 presents the most commonly established risk factors associated with the development of prostate cancer. According to the Centers for Disease Control and Prevention (CDC), the most common risk factor is age; the older the man, the greater the chance of getting prostate cancer [1]. A study of age-specific incidence curves reveals that prostate cancer begins to rise sharply after 55 years and peaks around the age of 70-74 years, declining slightly thereafter [12]. With age being the most common risk factor, race and ethnicity are also very important. The risk of prostate cancer is approximately 60% higher in African Americans than Caucasian males and is twice as likely to die from prostate cancer than other men [1,12]. African American males are also more likely to get prostate cancer at a younger age. Under such circumstances, cancer progression tends to be more severe by the time it is discovered [1]. Gann (2002) states that it is controversial whether the increased mortality rate amongst African American males is due to a difference in socioeconomic status and diagnosis stage or an inherent biological difference between the racial/ethnic groups [12]. About 10% to 15% of the difference in prostate cancer between the different ethnicities has been associated with differences in saturated fat intake [5]. A high-fat diet in the western world also helps explain why prostate cancer is more prevalent in developing countries than in Asia. Furthermore, high calcium intake has been associated with advanced prostate cancer; diets high in saturated fat and milk products also seem to increase the risk [6,13]. There is some evidence that suggests that a vegetarian diet may decrease the risk of prostate cancer. However, whether or not this is significant in value is yet to be determined [14].

Epidemiological studies conducted as far back as the 1950s determined that having a first-degree relative with prostate cancer increased the risk for an individual approximately two- to three-fold [1,12]. The risk is also further elevated by an early age of onset in relatives with the disease [1]. While family history is considered a significant risk factor in developing prostate cancer, the exact role of genes is undetermined. That being said, 6 genes have been said to be associated with prostate cancer: BRCA1, BRCA2, HPC1, HPC2, HPCX, and CAPB [12,15]. As presented in Figure 2, testosterone levels in men and its association with prostate cancer were investigated. In the early 1940s, researchers Charles Brenton Huggins and Clarence Hodges discovered that when a man’s testosterone levels declined, such as in old age, their prostate cancer stopped growing [15]. This finding would suggest that prostate cancer would be more common in the younger ages as testosterone levels tend to decrease with age. However, this finding is incongruent with trends and statistics noted by the CDC [1,16]. Furthermore, a 2016 meta-analysis of research found no relationship between a man’s testosterone levels and his risk of developing prostate cancer [17].

Androgen receptor (AR) plays an integral role in prostate cancer. However, the exact molecular mechanism for this androgen dependence has not yet been determined. In normal prostate epithelium, the function of AR is to stimulate the expression of proteins that are considered essential for the production of seminal fluids. AR regulates the transcription of multiple genes in the prostate and plays a central role in prostate cancer development. Most prostate cancers express a high level of AR and, thus, respond to androgen deprivation therapies [18]. Testosterone is the most abundant androgen in males. However, in the prostate, testosterone is converted into a more potent form called dihydrotestosterone (DHT) through the action of a resident enzyme known as 5-alpha reductase [19]. Embryologically, DHT is an important factor that is responsible for the development of the prostate gland. In the developing gland, AR acts as an important differentiation factor that is a prerequisite for prostate function and maintenance [20]. Studies have shown that androgens can stimulate the expression of G1 cyclins and cyclin-dependent kinases (CDKs) and decrease the expression of CDK inhibitors; however, the mechanism by which AR stimulates the cell cycle progression in prostate cancer cells remains unclear [18]. Though the exact role of AR in the development of prostate cancer is uncertain, androgen ablation therapies have been associated with triggering cell death or cell cycle arrest of prostate cancer cells [19].

Obesity is also a risk factor for the development of prostate cancer. Elevated body mass index (BMI) has been strongly correlated with prostate cancer mortality and has also been associated with low-grade progression to high-grade prostate cancer [6]. Obesity has been implicated in dysregulation of the insulin axis, inflammatory cytokine signaling, and induction of DNA-damaging oxidative stress, increasing the risk of several neoplasms, including breast, colorectal, and prostate cancers [21]. A study conducted by Kenfield et al. (2011) stated that risk for prostate cancer mortality was 1.6x greater amongst smokers, and cessation appeared to have a favorable effect on cancer progression, along with its mortality [21,22]. Much evidence suggests that obesity may promote the progression of prostate established prostate cancer rather than being a risk in the development of it [23]. With a Westernized, high fat diet also being a risk factor for the progression and development of prostate cancer, one can correlate obesity as a risk factor. The most abundantly consumed fatty aside in the Western diet is linoleic acid (omega-6 polyunsaturated fatty acid), which has recently been attributed to promoting prostate cancer migration in vitro [23,24]. In vitro studies have also shown that high levels of saturated fat act as a growth factor in various prostate cancer cell lines, whereas low-fat diets result in slower androgen-sensitive prostate cancer growth and can delay progression [24]. With a high-fat diet established as a risk factor for prostate cancer progression, the link between obesity and prostate cancer can also be assumed. The assumptive correlation between obesity and prostate cancer results from the high-fat diet that can ultimately lead to the progression of obesity. In theory, a Westernized, high fat diet can further obesity, and thus, obesity is linked as a risk factor for prostate cancer. It has been presumed that the circulating adipokines noted in obesity may actually be associated with prostate cancer. Specifically, adipokines' paracrine effects are important in cases of prostate cancer progression where extracapsular extension and invasion of the retropubic fat pad occurs [23,24].

Clinical Symptoms

The clinical symptoms noted in prostate cancer ultimately depend on whether the cancer is in the early stages or advanced stage and which lobe of the prostate gland is involved. It is also imperative to understand that while some individuals with prostate cancer may present with symptoms, others may be completely asymptomatic. It becomes imperative that the physician take the appropriate steps to do a thorough examination in such cases. The variety of symptoms may also differ if cancer has local spread or metastasis. In locally invasive prostate cancer, the transitional-zone tumors spread to the bladder neck, while the peripheral-zone tumors extend into the ejaculatory ducts and seminal vesicles [25]. 

**Figure 3:**
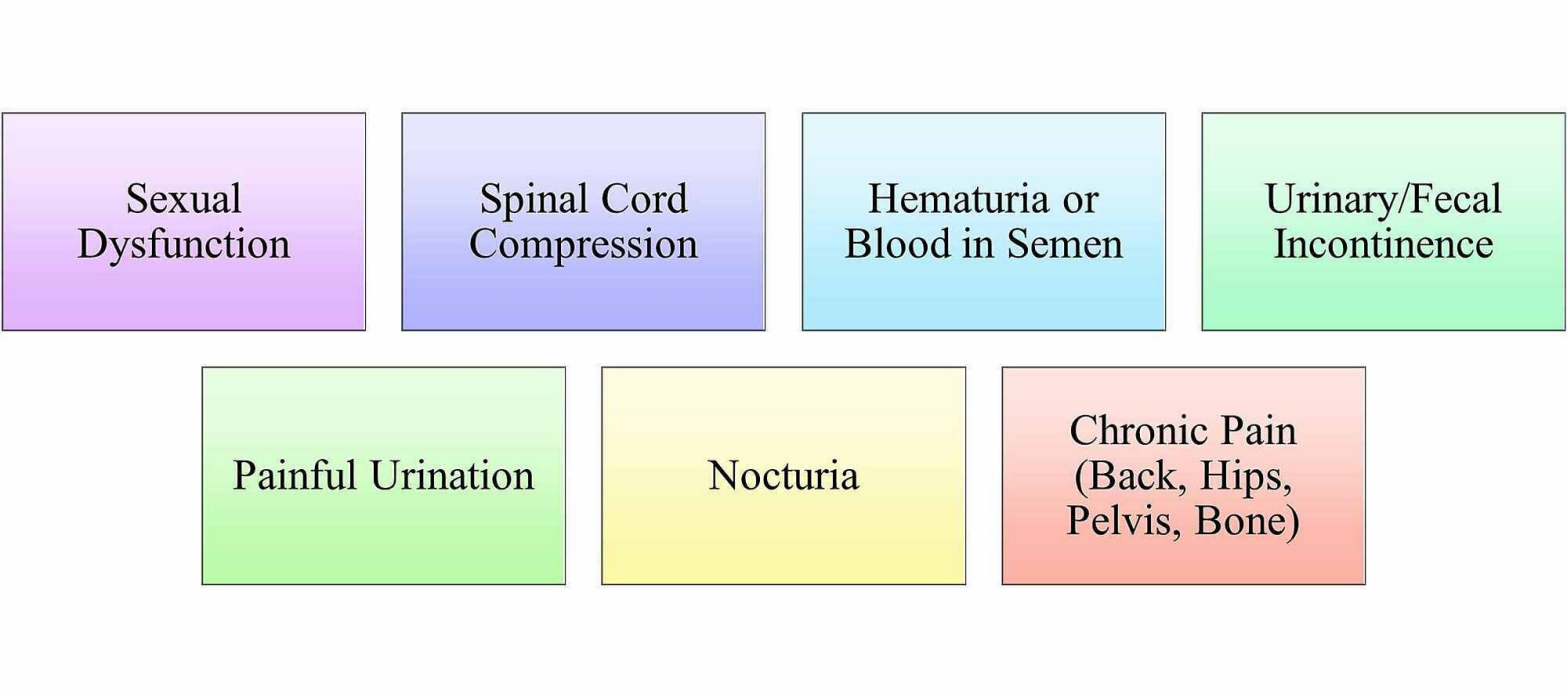
Symptoms associated with prostate cancer

Figure 3 provides a visualization of symptoms most commonly associated with prostate cancer. Chronic pain that radiates to the back, hips, or pelvis is associated with metastatic prostate cancer. The metastasis of prostate cancer to the vertebrae is also better known as Pott’s disease. This type of metastasis can further lead to the compression of the spinal cord. The spinal cord's compression would cause the individual to present with symptoms such as tingling or numbness of the lower extremities. Symptoms of urinary incontinence that mirror benign prostatic hyperplasia (BPH) can be noticed in the early stages of prostate cancer. Individuals may be coming in complaining of increased frequency of urination, painful urination, decreased force in the urine stream, or restless sleep due to increased urination throughout the night. In the later stages, symptoms such as sexual dysfunction may prevail, specifically erectile dysfunction and painful ejaculation. Painful ejaculation is due to the compression of the urethra within the prostate gland. It is the same compression of the urethra that causes an individual to present with painful urination. In extreme cases, an individual may present with hematuria or blood in the semen as well. In extreme cases, spinal cord compression may cause fecal incontinence as well. Metastasis to the bones is partially a result of the prostatic venous plexus draining into the vertebral veins [6].

Method of Diagnosis

Though PSA screenings' value remains controversial because it is non-specific, the prostate-specific antigen blood test is often the initial screening method utilized to detect any prostatic abnormalities [3,6]. The PSA array is not cancer-specific for prostate cancer. It is elevated in other situations such as benign prostatic hyperplasia (BPH), prostatitis, prostatic infarcts, sexual activity, and perineal trauma or infection [7,26]. Overall, the PSA sensitivity varies between 9% to 33% depending on the age and, essentially, this means that approximately 91% of individuals with elevated PSA levels do not have prostate cancer [26,27]. Although monitoring PSA levels may be controversial when it comes to prostate cancer diagnosis, it has been noted to be significantly helpful in determining how well the cancer is responding to therapy. Many physicians may also resort to conducting a digital rectal examination to feel for any abnormal contour or thickness to the prostate cancer. That being said, a DRE alone is not capable of detecting early cancer since not all types of cancers cause abnormal contours to the prostate in itself. In many cases, a DRE is combined with a PSA test, however, it also has low sensitivity and specificity. An abnormal DRE would result in findings of nodularity, induration, or asymmetry [7]. Prostate-specific antigen is a serine protease enzyme produced by the columnar epithelium of prostate cancer [26]. Locally, PSA prevents seminal coagulation by breaking down proteins such as semenogelin and fibronectin proteins responsible for the gel-like consistency of seminal fluid [26,28]. Breaking down semenogelin and fibronectin into smaller peptides helps in the process of impregnation [29]. Normally, there is a slight amount of PSA that leaks into the bloodstream, however, in any type of prostatic condition, PSA diffuses into the extracellular space and is then drained by the lymphatics into the bloodstream [7]. Any type of prostatic trauma causes damage to, and disrupts the gland's microarchitecture, ultimately leading to elevated PSA levels in the bloodstream [7,26].

While PSA's normal value is considered less than or equal to 4.0ng/ml, because serum PSA levels tend to increase faster in older men, age-specific ranges shown in Table 1 have been defined [7]. While age causes an increase in PSA, studies have shown that certain pharmaceutics has also been known to impact PSA levels: statins, thiazide diuretics, nonsteroidal anti-inflammatory drugs (NSAIDs), and 5-alpha-reductase inhibitors [6,7]. These specific medications have been noted to decrease PSA levels, and thus, a rise in PSA levels in a patient on these medications would evidently cause suspicion of prostate cancer [7,30]. The definitive method in diagnosing prostate cancer would be conducting a prostate biopsy. The decision on whether to conduct a biopsy is at the physician's discretion based on PSA and DRE results. Furthermore, the presence of certain risk factors may further impact a physician’s decision as to whether or not to move forward with conducting a biopsy. The biopsy is conducted through a transrectal approach and a transrectal ultrasound [1]. A transrectal ultrasound is when a probe the size of a finger is inserted into the rectum of an individual, and images of the prostate are taken [1,15]. The images taken via the ultrasound allow the healthcare professional to note the prostate gland's size and examine for any abnormalities. The healthcare provider will always pay attention to any areas of shadows within the prostate gland as these shadows might signal shadow, though this may not always be the case [15]. Not all shadows may be indicative of cancer. The ultrasound is also used to guide a needle towards the prostate. Antibiotics must be started the day before the biopsy and continued for three days following the biopsy to avoid any infectious complications [6]. Fluoroquinolones have been the most commonly used antibiotics for this purpose, but pre-biopsy rectal cultures are suggested to optimize prophylactic antibiotic selection [6,31]. The tissue sample obtained from the biopsy is then graded by using a Gleason grading system. The Gleason grade is based primarily on the architecture or arrangement of the malignant cells within the tumor and other factors such as degree of differentiation [31]. With this system, each tissue sample obtained during the biopsy is given a score between three and five [15,32]. The Gleason system will then combine the two most common grades that are found within the biopsied tissues and will provide the pathologist with a score; cancer with a score of six will be treated as low-risk cancer, scores around seven are treated as intermediate/mid-level cancers, and scores of eight or above are treated as high-risk cancers [15]. The Gleason score allows physicians to analyze with treatment options are available for the grade of cancer an individual may possess. A Gleason score of less than six usually indicates indolent cancer less likely to be clinically significant. A score of eight or greater is generally associated with poorly differentiated tumors that possess a worse prognosis [31,32].

**Table 1:** Age-specific PSA ranges PSA, prostate-specific antigen

Age Group (Years)	PSA Range
40 to 49	0 to 2.5 ng/ml
50 to 59	0 to 3.5 ng/ml
60 to 69	0 to 4.5 ng/ml
70 to 79	0 to 6.5 ng/ml

The first step in managing prostate cancer is determining whether any type of treatment needs to be initiated in the first place. Low-grade prostate cancer tumors typically grow very slowly and, thus, do not require any sort of intervention; this is especially true for particularly elderly patients and those with comorbidities that would reasonably limit life expectancy to ten additional years or less [6]. The most common approach of intervention in low-grade tumors is active surveillance. In the process of active surveillance, these patients are required to have regular PSA testing done and at least one additional biopsy 12 to 18 months following the original diagnosis [6,32]. Another type of surveillance, or lack thereof, is referred to as watchful waiting. Watchful waiting is a less involved system of monitoring cancer such that it does not involve regular biopsies or other active surveillance tools [15]. The largest risk with watchful watching is that treatment may become much more difficult if cancer progresses and spreads between follow-up visits or may no longer be a viable option altogether.

Pharmaceutical Treatment/Management

**Figure 4:**
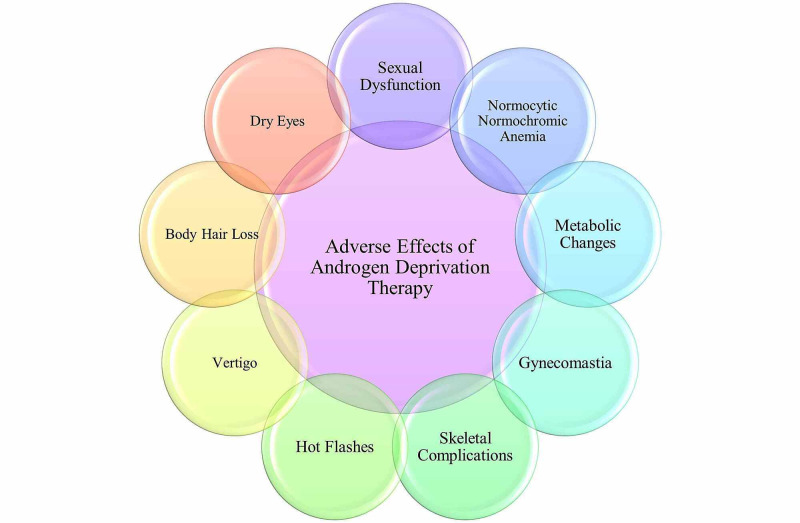
Adverse effects of androgen deprivation therapy

Different pharmaceutics can be administered to a patient, depending on whether the goal is to cure cancer or control/relieve some symptoms. A healthcare professional may administer any one of the medications listed in Table 2 or administer any combination. The inhibition of AR activity is the major therapeutic goal for managing metastatic disease [19]. Previous studies have shown that androgen deprivation causes a G0-G1 cell cycle arrest, whereas androgen stimulates proliferation and enhances the expression of multiple G1-S regulatory proteins [33]. Furthermore, prostatic adenocarcinomas respond very poorly to standard chemotherapy and for this reason, androgen ablation therapy remains the first line of intervention for metastatic conditions [20]. With the vast majority of the patients responding quite well to ADT, recurrent tumors have been noted to develop within a median time of two to three times; the development of such tumors is known as castrate-resistant prostate cancer (CRPC) [20,33]. Medical castration with gonadotropin-releasing hormone agonists (GnRH-As) in prostate cancer patients dates back to 1982 with leuprolide and goserelin the 2 most commonly used GnRH-As [34]. Long term treatment with GNRH-A supplants will affect the physiologically pulsatile endogenous GnRH and is thought to down-regulate its receptors in the pituitary gland, leading to castration levels of testosterone within three weeks [34,35]. In some cases, androgen receptor antagonists such as flutamide, bicalutamide, and nilutamide may be used either alone or in combination with castration to block the effects of androgens [34]. If advanced prostate cancer becomes androgen-independent following testosterone castration, secondary hormone treatment with androgen receptor antagonists or ketoconazole is used (Figure 4) [36].

**Table 2:** Pharmaceutical treatments currently approved for prostate cancer

Chemotherapy Drugs	Androgen Deprivation Therapy	Bone Metastasis	Radiopharmaceuticals
Cabazataxel	Abiraterone Acetate	Alendronate	Radium 223
Docetaxel	Apalutamide	Denosumab	
Mitoxantrone	Bicalutamide	Pamidronate	
	Buserelin Acetate	Zoledronic Acid	
	Cyproterone Acetate		
	Degarelix Acetate		
	Enzalutamide		
	Flutamide		
	Goserelin Acetate		
	Histrelin Acetate		
	Leuprolide Acetate		
	Triptorelin Pamoate		

A study conducted by Sharifi et al. (2005) outlines the various benefits and adverse effects of ADT [34]. Some of the quality of life benefits noted were reduced bone pain, pathological fractures, spinal cord compression, and ureteral obstruction [34,37]. Due to the nature of ADT to be transient in the sense that advanced prostate cancer always becomes androgen-independent following castration, the exact improvement in the long-term survival of patients is yet to be determined. Several adverse effects have also been associated with ADT. Up to 80% of patients undergoing treatment with GnRH-A have reported hot flashes [34]. In a clinical trial, megestrol acetate was investigated to prevent hot flashes in women with a history of breast cancer and men undergoing ADT for prostate cancer; results showed a reduction in hot flashes by 74% [34,38]. While megestrol has shown promising effects in clinical trials in reducing hot flashes, in men who commence megestrol therapy while concurrently taking ADT, PSA levels have been reported to increase. This transient increase in PSA levels has been noted to decrease upon the cessation of megestrol.

Skeletal complications, such as decreased bone mineral density (BMD), have been noted in men receiving ADT in prospective trials [34]. Men treated with ADT present an increased risk of fractures starting 1-year after the diagnosis [34,38]. Risk factors for osteoporosis should be evaluated in patients before beginning ADT therapy; family history, low body weight, prior fractures, excessive alcohol use, low vitamin D levels, and other medical comorbidities [34]. Calcium and vitamin D supplementation should be given concurrently with ADT therapy to minimize the risk of osteoporosis or other skeletal complications that may arise. Table 2 lists the most common bisphosphonates that have been utilized in conjunction with ADT therapy; however, this is not recommended unless there is documented osteoporosis or androgen-independent prostate cancer with skeletal metastasis [34, 39]. A randomized, placebo-controlled trial in patients with androgen-independent metastatic cancer showed a significant decrease in skeletal-related events when zoledronic acid was administered [34]. The greatest known adverse effect of bisphosphonate use is the jaw's osteonecrosis, and for this reason, the use of bisphosphonates is not a first-line intervention. Moreover, in situations where the medication benefits will outweigh the potential risks, the use of bisphosphonates may prove to be more advantageous than detrimental to the individual.

Sexual dysfunction is an adverse effect of ADT that is linked to the castration of testosterone. Testosterone plays a vital role in the normal sexual function of a male. Although erectile dysfunction is not uncommon after radical prostatectomy, men who undergo ADT have a further decline in sexual intercourse and a decrease in sexual desire than men who are not treated with ADT [34,40]. A decrease in sexual libido can distress the patient and place greater stress on any intimate relationships. Dealing with cancer can cause an individual to feel lonely, and the lack of being able to initiate or perform intimately may further cause feelings of inadequacy or alienation.

A study conducted by Strum et al. (2003) found that patients receiving androgen blockade treatment presented a decrease of at least 10% in hemoglobin in about 90% of their patients [41]. The presentation of normocytic normochromic anemia in these patients can further contribute to the presentation of fatigue. Gynecomastia is an adverse effect noted in about 1% to 16% of patients treated with ADT [34]. Though momentarily alarming for the patient, this adverse effect can be treated with medications such as tamoxifen or surgery, depending on the extremity of the condition. Other adverse effects noted in ADT include dry eyes, body hair loss, and vertigo. These adverse effects of ADT tend to mimic the presentation of testosterone deficiency.

Surgical Treatment/Management

Radical prostatectomy (RP) remains the cornerstone of curative prostate cancer treatment. It is deemed appropriate for men with localized prostate cancer with at least 10 years of life expectancy or any patient with high-risk prostate cancer [42]. A prostatectomy is a surgical procedure in which surgeons choose to remove just the prostate, however, in radical prostatectomy, the tissue surrounding the prostate is also removed. There are several different methods of RP; however, the most common approach is with a retropubic/suprapubic approach [43]. While the goal of RP is to remove all of the prostate cancer, this procedure is only used when the cancer is believed to be confined to within the prostate gland itself [43,44].

**Figure 5:**
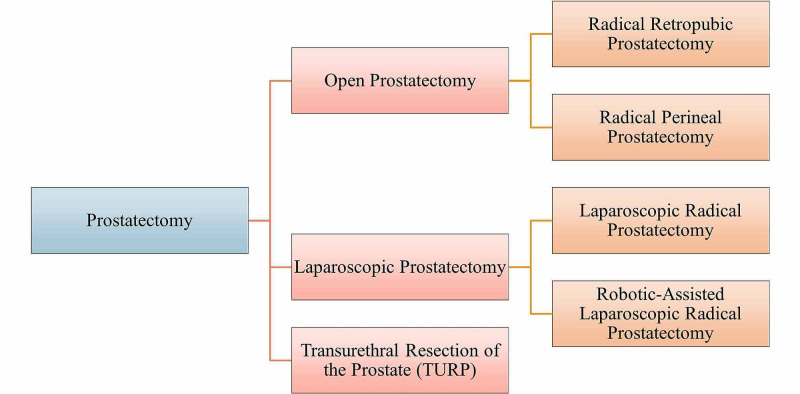
Different methods of prostatectomy

Figure 5 outlines the various methods of prostatectomy that may be utilized. In a radical retropubic prostatectomy, the surgeon will make an incision in the lower abdomen. In radical perineal prostatectomy, the incision is made in the skin fold between the anus and the scrotum - the perineum. Both radical retropubic and radical perineal prostatectomies are examples of open prostatectomies, which are more of a traditional approach and, thus, done less often than in the past [44]. With advancements in technology, there is a lesser need to make incisions and be as invasive. Open prostatectomies are more likely to warrant a longer stay in hospital settings, post-surgery, and require a greater amount of time allocated towards healing. Furthermore, according to Wroński (2012), the risk of wound infection is approximately 5% to 9% in open prostatectomy procedures [45]. If there’s reason to believe that cancer has spread to the lymph nodes, the surgeon will also go ahead and remove the lymph nodes in the vicinity of the prostate gland [43,44]. If cancer has spread to the lymph nodes, it suggests cancer has spread beyond the prostate and the surgeons may decide to discontinue the surgery as it will not be enough to treat cancer properly [44]. Additional treatments may be utilized alongside the prostatectomy to ensure that the metastasis is eradicated. Following an open prostatectomy, a catheter is placed to drain the bladder and is removed after one to two weeks. Among the two types of open prostatectomy methods, a radical perineal approach is more likely to cause erection problems and is used less often [43,44]. Another reason why a radical perineal approach is made less often is that lymph nodes' resection is not possible through this approach. Overall, a radical perineal approach is less painful and an easier recovery than a retropubic approach. The healing time following the surgery varies to be about the same.

The other branch of methods that could be used is laparoscopic. With a laparoscopic procedure, smaller incisions are made, however, multiple may be made for the surgeon to insert the instruments appropriately. Overall, multiple benefits outweigh the open prostatectomy approach. These include faster recovery times, less blood loss, and pain and the catheter can be removed earlier on than with open prostatectomies [44]. Wroński (2012) states that the risk of wound infection in laparoscopic radical prostatectomies is approximately 1% [45]. For the most part, a laparoscopic radical prostatectomy has been replaced by a robotic-assisted laparoscopic radical prostatectomy (RALP): the most common type of prostate cancer surgery done today [3]. RALP is done using a robotic system in which the surgeon sits at a control panel in the operating room and moves the robotic arms to operate through small incisions made in the patient’s abdomen [3,44]. The robotic system essentially provides more maneuverability and a greater level of precision when moving the instruments for the surgeon. However, the most important factor in the success of either type of laparoscopic procedure does come back to the surgeon’s experience and skills [3,43,44].

Transurethral resection of the prostate (TURP) is more commonly used in treating men with non-cancerous enlargement of the prostate, such as in benign prostatic hyperplasia [44]. In this procedure, the surgeon will remove the part of the prostate gland proximal to the urethra, which is done via a resectoscope [43,44]. No incisions are made in this procedure because the resectoscope is passed through the tip of the penis and into the urethra to the point where it is running through the prostate gland. Once the resectoscope is in position, either electricity or laser is passed through to heat it, or a laser is used to cut or vaporize the tissue [44]. TURP does not cure cancer, but it can remove obstructions to provide temporary relief while urologists can develop a more definitive plan. TURP may be utilized to essentially relieve the patient of symptoms such as urinary obstruction or nocturia, or frequent urination.

Despite the type of procedure done, Wroński (2012) notes that the list of adverse effects of a prostatectomy seems to remain the same [45]. The only thing that differs is that when doing a radical prostatectomy, the entire cancer is removed. This is true as long as cancer has not spread outside the prostate [3]. In other approaches, specifically laparoscopic, lymph nodes are not easily accessible, and not all of the cancer may be removed, which will create the possibility of relapse. In some cases, a laparoscopic approach may be followed with pharmaceutical interventions such as androgen deprivation therapy to eradicate cancer that was not removed.

**Figure 6:**
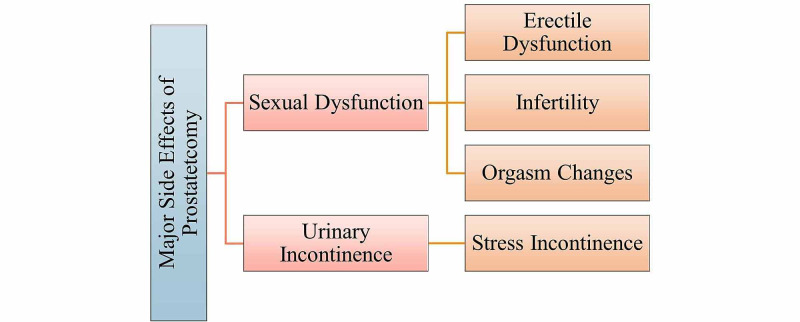
Major side effects of prostate surgery

The two most common and major side effects of all prostate surgery approaches are listed in Figure 6. Urinary incontinence is defined as the inability to control urinary flow, and thus, a patient may present with leakage or dribbling [44]. Stress incontinence is the most common type of incontinence that patients may present with following prostate surgery. Stress incontinence is defined as urine leakage when a patient cough, laughs, sneezes, or is exercising [3]. Urinary incontinence can impact an individual’s quality of life, emotionally and socially. An individual may not present with frequent urination throughout the night; however, he may complain of leakage in the morning upon getting out of bed. Sexual dysfunction is another side effect of prostate surgery with the main side effect being that of erectile dysfunction. Erectile dysfunction, also referred to as impotence, is defined as a man's inability to have an erection long enough for sufficient sexual penetration [3,44]. Erections are controlled by bundles of nerves that run adjacent to either side of the prostate. If the patient can have erections before surgery, the surgeon will attempt to save the nerves in something referred to as a nerve-sparing approach. If cancer has grown very close to, or into the nerves, the surgeon will need to remove the nerves. If only one nerve bundle is removed, the patient may be able to have erections. Still, a lower chance of being able to, however, if both nerve bundles are removed, the patient will not have spontaneous erections [44]. If no nerve bundles are removed, the patient will have normal erections before the surgery. Because an erection is not necessary for a male to have an orgasm, men will be able to have an orgasm following radical prostatectomy [3]. The orgasmic changes a male may come across are a decrease of fluid with an orgasm. The decrease in the fluid is largely attributed to removing the prostate gland, seminal vesicles, and the division of the vas deferens during the surgery [3,45]. Because there is a lack of appropriate seminal fluid in the orgasm, patients cannot cause a pregnancy following prostate surgery. Sperm requires the enzymes within the seminal fluid to allow appropriate motility and successful fertilization, and thus, patients may present with infertility following radical prostatectomy.

## Conclusions

Despite high incidence rates of prostate cancer in the United States, the mortality rates continue to remain low due to screening methods that allow for early intervention in patients. Even with low mortality rates, prostate cancer still remains one of the leading causes of death amongst men. One in every nine men in the United States will be diagnosed with prostate cancer at some point in their life. The prostate gland is located inferior to the bladder and anterior to the rectum and has two smaller glands attached on either side that is known as the seminal vesicles. Due to the close proximity of the seminal vesicles to the prostate gland, cancer may occasionally spread to the seminal vesicles and may also be removed during radical prostatectomies. There are several risk factors that have been associated with prostate cancer, with age being the greatest risk. The likelihood of men developing prostate cancer increases as they age. Prostate cancer has also been associated with being more common in the Western world. Prostate adenocarcinoma is the most common type of prostate cancer seen, however in rare cases, other types may also be presented. Whether there is any genetic variability amongst the different types of prostate cancers requires further study. Radical prostatectomies are considered the ultimate treatment of cancer as the surgeon is able to completely take out cancer and the surrounding tissues. The type of intervention used is dependent on the grading and extensive nature of cancer. in the event that cancer has spread to the lymph nodes near the prostate gland, the surgeon may decide to either remove the lymph nodes or completely discontinue the prostate surgery. The discontinuation of the surgery may be a decision that is made on the basis of metastasis has already occurred, in which cases palliative methods may be introduced to the patient. Some surgical interventions may be paired alongside pharmaceutical interventions to aid in the complete eradication of cancer. Both types of interventions present with risks and adverse effects and more research need to occur in attempting to understand how the side effects of these approaches can be further minimized.
